# Green Analytical Approach for the Determination of Zinc in Pharmaceutical Product Using Natural Reagent

**DOI:** 10.1155/2022/8520432

**Published:** 2022-04-23

**Authors:** Hemraj Sharma, Arjun Sharma, Bimala Sharma, Sonu Karna

**Affiliations:** Department of Pharmacy, Shree Medical and Technical College, Bharatpur, Chitwan, Nepal

## Abstract

A selective, sensitive, and environmentally safe spectrophotometry method was developed and validated for the determination of zinc in pharmaceutical substances using natural reagents obtained from the leaves of plant *Acacia* catechu. Different factors were optimized such as volume of reagent, selection of pH, and stability of the color complex. The drug showed a stable yellowish orange color complex at 550 nm. The greenness of the methods was estimated using an eco-scale tool where the presented method was found to be excellent green with an ecoscore of 84 based on spectrophotometric determination. Also, the greenness of the method was assessed by the Green Analytical Procedure Index and found to be eco-friendly. The method was validated in conformance with ICH guidelines, with acceptable values for linearity, accuracy, precision, LOD, and LOQ. The linearity range for zinc sulphate was 5–25 *μ*g mL^−1^ with an *R*^2^ value of 0.996. The % RSD for intraday precision and interday precision was less than 2%. The suggested method can be employed for the economic analysis of zinc in its pure form and various formulations. The presented spectrophotometric method is the first analytical method for the analysis of zinc present in zinc sulphate and showed greater ecoscale as compared to the official method.

## 1. Introduction

Zinc is the second most essential trace element in all residing structures, from animals to humans, and performs a crucial function in lots of metabolic procedures in the body [1]. Each day's nutritional consumption of zinc is crucial to adjust the cell division through regulating the synthesis of protein and DNA [2]. The importance of zinc in human nutrients and public health has become recognized distinctly recently. Zinc insufficiently has been diagnosed by some professionals as a crucial public health issue, particularly in growing countries. The prevalence and medical results of zinc deficiency consist of growth delay, diarrhoea, pneumonia, disturbed neuropsychological overall performance, and abnormalities in foetal development [3]. Zinc deficiency has been observed in lots of illnesses which include sickle cell anaemia, kidney disease, and gastrointestinal disorders. It also can make contributions to disruptions inside the immune system that accompany those diseases. In fact, a current study at Michigan State University in East Lansing suggests that the immune structures of animals given good enough zinc for a month reduce ineffectiveness by30 to 80%. [4]. In the current scenario, zinc emerges as an essential element of preference for the control of COVID-19 signs and symptoms in conjunction with anti-infective and different antiparasitic drugs [5]. Few analytical techniques have been mentioned for the assay of zinc, which encompass spectrophotometric methods [6–8], HPLC [9, 10], polarography [11, 12], potentiometry [13–17], flame AAS [18–23], and mass spectrometry [24–31].

The literature review revealed that the complexometric titration is used for the analysis of zinc (zinc sulphate) through pharmacopoeial techniques [32], wherein huge quantities of organic compounds such as hexamine and acetic acid are used. It is a destructive technique and makes use of a big amount of sample being analyzed, big chemical waste, limited accuracy, and greater risk of human error. Similarly, the stated different techniques are afflicted by lack of sensitivity and consume huge quantities of organic solvents that harm the surroundings and raise the cost. HPLC and different electrochemical techniques such as polarography and potentiometry are the most regularly used and well-advanced techniques for both qualitative and quantitative analysis. However, most of the electrochemical and chromatographic applications use hazardous solvents and generate big quantities of poisonous waste that impact the surroundings. Recently, the Society of Analytical Chemistry has been growingly in search of the implementation of green techniques that take away or lower poisonous and corrosive waste [33]. Evaluation of analytical techniques greenness is profoundly critical and highly interesting to maximum method developers. One of the modern day used greenness valuation tools for analytical strategies is the analytical ecoscale [34] that is a semiquantitative tool to assess and evaluate analytical techniques primarily based totally on their conformity with green chemistry principles [35]. The ecoscale tool reflects many elements that may have unfavourable influences on the environment, which include the magnificence and quantity of any chemical used within side the procedure, the mass of generated waste, occupational exposure, and power consumption [36]. UV spectrophotometry has huge applications within side the area of pharmaceutical analysis, both for identity and for quantitative analysis of various drugs. The principal purpose of the existing study is to establish a tremendously simple, sensitive, legitimate, and cheaper green analytical method for the determination of zinc sulphate within side the pure form using natural reagents received from *Acacia* catechu which limits the use of chemicals and is a cost-effective technique. The green analytical technique is a fast, economical, and environment friendly technique. It might be an amazing alternative technique of extraction that allows decreasing the usage of chemical reagent which might assist to lessen the waste and allows the use of renewable sources.

## 2. Materials and Methods

### 2.1. Material

#### 2.1.1. Plant Material

The dried leaves of *Acacia* catechu were used for the extraction procedure of polyphones.

#### 2.1.2. Pure Sample and Chemicals

Zinc sulphate, methanol, acetone, and sodium dihydrogen phosphate were purchased from Merck Life Science Pvt. Ltd. (Mumbai).

#### 2.1.3. Equipment Required

LT-2100 Double beam UV-visual spectrophotometer with a 10 mm quartz cuvette was used to record the absorbance.

### 2.2. Methodology

#### 2.2.1. Collection of *Acacia* Catechu

Leaves of *Acacia* catechu were collected from Salyan and Dang, Nepal. Leaves were shade dried and homogenized into the form of powder.

#### 2.2.2. Extraction

*Acacia* catechu leaves after collection were cleaned, washed with deionized water, dried at room temperature, and then cut into small pieces and shade dried. The dried leaves were ground to fine powder form using a blender machine and then homogeneously mixed and stored in a dry place until required for use. The extraction of 1 kg of dried powder of the *Acacia* catechu leaves was carried out by a cold maceration process using various solvents such as water, methanol, and acetone. The extracts were then filtered through muslin cloth and Whatman filter paper. The filtrates were concentrated under reduced pressure to obtain residues. After evaporation, the extractive value for each extract was calculated by using the following formula:

(1) $$\begin{array}{c} \frac{\text{\% extraction value}\left(\text{w}/\text{w}\right)}{\text{\%yield}}=\frac{\text{weight of crude extract}}{\text{weight of dried plant part used }}\times 100\text{.} \end{array}$$

### 2.3. Preparation of Natural Reagent

**Figure 1:**
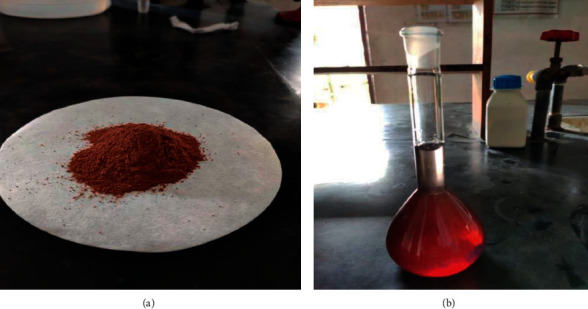
(a, b) Dried powder and solution of natural reagent *Acacia* catechu.

Plant powder (50 g) and deionized water (100 mL) were stirred in an extraction flask for 30 minutes. The extract was filtered through a filter paper and made up to a volume of 100 mL with de-ionized water. The preparation of the dried powder of *Acacia* catechu and reagent is shown in Figure 1(a) and Figure 1(b), respectively.

### 2.4. Method Development

The proposed method was developed as per Kumar Reddy et al. [37].

#### 2.4.1. Selection of Suitable Wavelength

The wave length maximum was selected by varying the wavelength from 400 nm to 800 nm and reading each absorbance value of every wavelength in the spectrophotometry directly where 550 nm gave the maximum absorbance value and was selected as the desired wavelength.

#### 2.4.2. Selection of Suitable pH

The pH was selected by making the acetate buffer pH 3.7 to 6. The pH of the acetate buffer to be added was optimized.

#### 2.4.3. Selection of Reagent Volume

The volume optimization was carried out by varying the volume from 2–5 mL.

#### 2.4.4. Mechanism of Coloured Production

**Figure 2:**
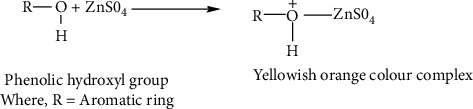
Formation of colour complex.

Zinc sulphate and natural reagent obtain from *Acacia* catechu were allowed to react to form the colour complex, as metal ion (zinc) reacts with ligand molecule (plant's polyphenols) to form colour complex. *Acacia* catechu contains phenolic hydroxyl groups which react with zinc sulphate to form a yellowish orange colour complex. The idea of color development was taken from Siriangkhawut et al. [38], with some modifications where instead of iron, zinc was taken as the metal of analysis. The possible reaction for the development of colour is described in Figure 2.

### 2.5. Phytochemical Screening Test

Various phytochemical tests were performed for the fresh fruit extracts and marketed juices. The samples were tested for the presence of active principles such as triterpenoids, glycosides, alkaloids, saponins, carbohydrates, flavonoids, tannins, phenols, vitamin C, and protein. The phytochemical screening was performed by following standard procedures [39, 40].

### 2.6. Determination of Total Phenol

The Folin–Ciocalteu method was used for the determination of total phenol [41]. The total phenol content is expressed as milligram of gallic acid equivalent per gram of dry extract weight using the calibration curve of gallic acid (50 mg·L^−1^–400 mg·L^−1^) standards.

### 2.7. Sample Preparation

Four different formulations of zinc sulphate were used and 20 tablets from each formulation were used and powdered and average weight of powder was taken and dissolved in a 100 ml volumetric flask and the filtered concentration was made up to 9 *μ*g·mL^−1^.

## 3. Result and Discussion

### 3.1. Extraction of Natural Reagent from *Acacia* Catechu Leaves

The main components in *Acacia* catechu leaf are phenolic compounds [42]. The type of solvent has a strong impact on the yield of extraction. In this study, different polar solvents such as water, methanol, ethanol, and acetone were investigated. The total phenolic content (as tannic acid) of each extract was evaluated. The methanol extract gave the highest tannic acid (8.3 g 100 g^−1^). The total phenolic contents (as tannic acid) for water and acetone were 7.18, and 3.94 g 100 g^−1^, respectively. Water extract of the leaves yields an appreciable amount of phenolic compounds which is nearly equal to methanol. Hence, to make it green and increase its analytical ecoscale value, water extract was used for the rest of the analysis instead of methanol.

### 3.2. Optimization of Method

A natural reagent obtained from *Acacia* catechu was allowed to react with zinc sulphate along with acetate buffer to form a reddish product with an absorption maxima of 550 nm. The optimization of the research was first established by varying the volume of reagent (2 to 6 mL), selection of pH, and stability of the complex.

#### 3.2.1. Optimization of Reagent Volume

The optimization of the research was first established by varying the volume of reagent (2 to 6 mL), where we found maximum absorbance at 5 mL. Hence, it was selected as optimized volume of the reagent, as shown in Table 1.

#### 3.2.2. Optimization of Reaction Stability

After optimizing the volume of reagent, the drug (zinc sulphate) and reagent were mixed to develop the colour. The optimum time for completion of the reaction between zinc sulphate and natural reagent obtained from *Acacia* catechu was 1 min, and the colour complex was stable for 6 hours (Table 2) and absorbance was measured. It was quite stable with precise measurement.

#### 3.2.3. Optimization of pH

After optimization of the volume of reagent, the acetate buffer of pH 3.7 to 6 was made and absorbance was observed. On increasing the pH from 3.7 to 6, there was a corresponding increase in absorbance up to pH 5, but at pH 6, turbid solutions were observed. Amongst all, pH 5 gave the maximum absorbance and it was selected as the optimized pH for the analysis which is shown in Table 3.

### 3.3. Phytochemical Analysis

The phytochemical screening was carried out on *Acacia* catechu leaves, and the results are shown in Table 4. The color development in this method was possible only due to the positive test for phenols.

### 3.4. Estimation of Total Phenolic Content

**Figure 3:**
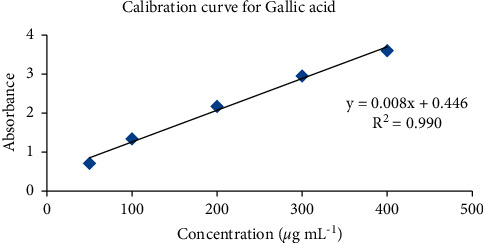
Calibration curve for gallic acid.

The total phenolic content, determined by the Folin–Ciocalteu method, gives an idea about the plant's phenolic hydroxyl groups reacting with the Folin–Ciocalteu reagent. The standard gallic acid calibration curve (50 *μ*g·mL^−1^–500 *μ*g·mL^−1^) was used to estimate the total phenol content of the extracts, as shown in Figure 3. The content of phenolic compounds in plant extracts was found to be 35.25 *μ*g·mL^−1^.

### 3.5. Method Validation

Validation of the proposed spectrophotometric method was performed according to ICH guidelines [43].

#### 3.5.1. Linearity

**Figure 4:**
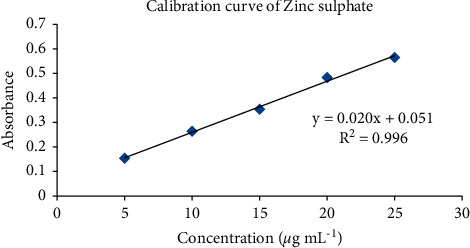
Calibration curve of zinc sulphate.

The colored complex of the zinc sulphate with natural reagent was analyzed using a UV-Visible spectrophotometer and the absorbance of all multiple standards was taken to construct a calibration curve, as shown in Figure 4. The linearity with the regression plot in the concentration range of 5–25 *μ*g·mL^−1^ showed a good relation with a correlation coefficient (*R*^2^) of 0.996.

#### 3.5.2. Precision

The data for intraday and interday precision for absorbance studies were obtained from three different concentrations of 6, 9, and 12 *μ*g·mL^−1^ in linearity. The % RSD values for intraday and interday precision were less than 2, and the result is as shown in Table 5.

#### 3.5.3. Accuracy

The accuracy of the method was evaluated in triplicate at three concentration levels, i.e., 80%, 100%, and 120% of the test concentration (9 *μ*g·mL^−1^). The percentage of recoveries was calculated and shown in Table 6.

#### 3.5.4. Limits of Detection and Qualification

The limit of detection (LOD) and limit of qualification (LOQ) for the procedure were performed on samples containing concentrations of LOD (0.770 *μ*g·mL^−1^) and LOQ (2.33 *μ*g·mL^−1^), respectively.

#### 3.5.5. Robustness

The robustness of the proposed method was also determined by changing the *λ* max of the analysis (*λ* max 550 nm) by ±1.0 nm and % mean recovery was reported which was found to be in between 98.9–101.95 ± 0.008, indicating it to be a sufficiently robust method.

#### 3.5.6. Assay

The assay procedure was performed in triplicate and the percentage of drugs found in the formulation and the mean and standard deviation in the formulation were calculated and are shown in Table 7.

### 3.6. Greenness Profile Evaluation of the Proposed Spectrophotometric Method

#### 3.6.1. Assessment Using Analytical Ecoscale

The analytical eco-scale assessment is an excellent semiquantitative method applied to assess the greenness profile of the analytical methods. According to penalty points, the total score of the method is calculated. The perfect green method has an ecoscale score of 100. Excellent and fair green methods have total scores of more than 75 and 50, respectively. If the method has a score of less than 50, it is called the deficient green method [44]. The ecoscale score of the introduced method is 84, as detailed in Table 8.

Penalty points have been established for every of the 4 main parameters of the analytical technique that depart from the appropriate green analysis: the quantity of solvents, hazardousness, energy depletion, and waste production penalty points for hazards rely upon the Globally Harmonized System of Classification and Labeling of Chemicals (GHS). The hazardous properties of the reagents have been labelled primarily based totally on GHS class as “danger” or “warning” via one or greater of 9 graphical pictograms assigned via GHS [34]. The analytical ecoscale rating was established for the recommended spectrophotometric technique and the official complexometric technique [32] and evaluation was carried out among them, as proven in Table 8. It explored that the proposed approach had a score of 84, and hence it proved to be a tremendous green analytical technique. Unlike the official one [32], which had a score of 66, taking into consideration fair green analytical technique.

#### 3.6.2. Assessment Using Green Analytical Procedure Index (GAPI)

GAPI [45, 46] is another qualitative method, used to measure the greenness based upon the stages involved in an analytical method. GAPI focuses on two aspects: (I) sample preparation and (II) instrumentation assessment. The GAPI tool uses a pictogram to classify the greenness of each stage of an analytical procedure, using a color scale with three levels of evaluation for each stage. In GAPI, a specific symbol with five pentagrams is used to evaluate and quantify (from green through yellow to red) the low, medium, and high environmental impact involved for each step of the methodology. Each field reflects a different aspect of the described analytical procedure and the field is filled green if certain requirements are met.

**Figure 5:**
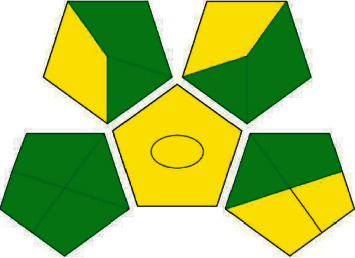
Assessment of proposed method by GAPI.

The application of GAPI in the proposed method is given in Table 9, and the pictogram is represented in Figure 5, which shows the method has satisfied most of the criteria and confirms the proposed method as eco-friendly.

## 4. Conclusion

The presented spectrophotometric method is the first analytical method for the analysis of zinc in zinc sulphate. Its ability for quantification of the studied drugs in pharmaceutical products gives it the advantage of being an excellent green, sensitive, and cost-effective spectrophotometric method.

Being aliphatic drug, assessment of zinc sulphate was a great challenge which was resolved by simple derivatization method using natural reagent. Validation of the proposed method followed ICH guidelines, allowing application of the suggested method for determination of the studied drugs in pharmaceutical products. Moreover, ecological evaluation of the presented spectrophotometric method using the ecoscale, GAPI assessment method affirmed its ecosafety.

## Figures and Tables

**Table 1:** Optimization of reagent volume.

Volume of reagent (mL)	Absorbance
2	0.224
3	0.268
4	0.291
5	0.331
6	0.281

**Table 2:** Stability of colour complex.

Time (hours)	Absorbance
1	0.522
2	0.492
3	0.438
4	0.412
5	0.408
6	0.402
7	0.268

**Table 3:** Selection of pH.

pH	Absorbance
3.7	0.104
4	0.202
4.6	0.222
5	0.318
6	0.231

**Table 4:** Phytochemical screening of *Acacia* catechu.

Phytochemical test	Test performed	Inferences	Result
Alkaliods	Mayer's test	Pale yellow ppt	+
	Wagner's test	Brown ppt	+
Flavanoids	Alkaline reagent test	Intense yellow colour	−
	Lead acetate test	Yellow colour ppt	−
Tannins	Ferric chloride test	Brownish green	+
Phenol		Blue, green, red, or purple color	+
Glycosides	Keller Killiani test	Blue colour in acetic lyarer	−
	Legal test	Blood red colour	−

*Note*. “+” sign indicates the presence, and “−” sign indicates the absence of phytochemical.

**Table 5:** Intraday and interday precision.

S.No.	Amount (*μ*g·mL^−1^)	Intraday absorbance (*n* = 3)		Interday absorbance (*n* = 3)	
		Amount found Mean ± SD	% RSD	Amount found Mean ± SD	% RSD
1	6	0.305 ± 0.005	1.31	0.305 ± 0.005	1.69
2	9	0.343 ± 0.005	1.31	0.343 ± 0.005	1.29
3	12	0.382 ± 0.006	1.46	0.382 ± 0.006	1.56

**Table 6:** Accuracy for zinc sulphate.

S.No.	Name of the drug	Amount of drug (*μ*g mL^−1^)	Recovery level (%)	Amount of drug added (*μ*g mL^−1^)	Amount found *μ*g mL^−1^ (Mean ± SD)	% Recovery (*n* = 3)
1.	Zinc sulphate	9	80	7.2	16.06 ± 0.25	99.00
			100	9	17.85 ± 0.13	99.16
			120	10.8	19.75 ± 0.23	99.74

**Table 7:** Assay for zinc sulphate.

S.No	Formulation^a^	Labelled claim (mg)	Amount found (Mean ± SD)	Assay (%)
1.	Tablet-1	10	8.95 ± 0.05	99.44
2.	Tablet-2	10	9.09 ± 0.05	100.55
3.	Tablet-3	20	9.033 ± 0.07	100.36
4.	Tablet-4	20	9.2 ± 0.05	101.89

^a^Tablet-1 to Tablet-4 are four marketed formulations from different companies.

**Table 8:** Penalty points for the determination of zinc by proposed spectrophotometric method and the official complexometry method [32].

Reagent/instruments	Penalty points	
	Proposed spectrophotometric method	Official complexometry method
HCl	4	−
Sodium EDTA	−	6
Sodium hydroxide	2	−
Acetic acid	−	8
Sodium chloride	2	−
Hexamine	−	4
Ferric chloride	2	−
Sodium carbonate	0	2
Water	0	0
Ammonia solution	0	6
Phosphate buffer	0	−
Occupational hazards	0	0
Waste	6	8
Instruments energy	0	0
Total penalty points	Σ16	Σ34
Analytical ecoscale total score	84	66

Comment	Excellent green analytical method	Fair green analytical method

**Table 9:** Assessment of GAPI for the proposed method.

S.No.	Category	Proposed method	Colour
I	Sample preparation		
1	Collection	UV	Green
2	Preservation	None	Green
3	Transport	None	Green
4	Storage	None	Green
5	Type of method: direct or indirect	Simple procedures	Yellow
6	Scale of extraction	Simple extraction using water	Green
7	Solvents/reagents used	Green solvents	Yellow
8	Additional treatments	None	Green
9	Reagent and solvents amount	<10 mL	Green
10	Health hazard	HCl and NaOH	Yellow
11	Safety hazard	HCl and NaOH were used very less so that flammability will be negligible.	Green

II	Instrumentation		
12	Energy	UV consumes ≤0.1 kWh per sample	Green
13	Occupational hazard (OH)	None	Green
14	Waste	Waste generated by the proposed method were 1–10 mL.	Yellow
15	Waste treatment	Low degradation	Yellow

Additional mark: quantification ring in the middle of GAPI: procedure for quantification.

## Data Availability

The data used to support the findings of this study are included within the article.
